# The Effect of Whole-Body Traditional and Functional Resistance Training on CAVI and Its Association With Muscular Fitness in Untrained Young Men

**DOI:** 10.3389/fphys.2022.888048

**Published:** 2022-05-25

**Authors:** Chongwen Zuo, Shumin Bo, Qing Li, Li Zhang

**Affiliations:** ^1^ Graduate Department of Capital University of Physical Education and Sports, Beijing, China; ^2^ School of Kinesiology and Health of Capital University of Physical Education and Sports, Beijing, China

**Keywords:** cardio-ankle vascular index, resistance training, muscular fitness, cardiovascular diseas, systemic arterial stiffness

## Abstract

**Background:** Resistance training-induced changes in the muscle function is essential for the health promotion of the young and older, but the discrepancies of the effect of resistance training on arterial stiffness leads to the divergence regarding to the effect of resistance training on cardiovascular health. What confuses our understanding in this field may be the following factors: external load (higher intensity vs. lighter intensity), participants’ cardiovascular health, and arterial stiffness assessment measurement. The purpose of the present study was to investigate the effects of the whole-body traditional high-intensity vs. functional low-intensity resistance training protocol on systemic arterial stiffness, and their association with muscular fitness components in untrained young men.

**Methods:** In this randomized controlled trial, twenty-nine untrained young men (mean age about 22.5 years old) were randomized into a 6-weeks (three sessions per week) supervised whole-body traditional high-intensity resistance group (TRT, *n* = 15) consisting of 4–5 sets of 12 repetitions (70%1RM, lower-repetitions) or a whole-body functional low-intensity resistance group (FRT, *n* = 14) with 4–5 sets of 20 repetitions (40%1RM, higher-repetitions) to volitional failure. The systemic arterial stiffness (cardio-ankle vascular index, CAVI) and muscular fitness components were assessed before and after the 6-weeks training program.

**Results:** There was a significant decrease (pre-post) for CAVI only in FRT group (*p* < 0.05), but no significant difference was observed between two groups. In addition, the TRT and FRT groups showed equally significantly increased in maximal strength, muscular endurance and power (within group: both *p* < 0.01); however, the independent *t* test exhibited that the difference between two groups in terms of change in maximal strength, muscular endurance and power were no significant (*p* > 0.05). Furthermore, the reduction in CAVI was negatively correlated with the increase in 1RM of bench press for all participants (r = −0.490, *p* < 0.01).

**Conclusion:**Using present criterion-standard assessments measurements demonstrates that CAVI was significantly reduced after 6-weeks functional resistance training with beneficial effect on muscular fitness. Negative and significant association between CAVI and 1RM bench press indicated the cardiovascular health may be involved in the regulation of resistance training.

## Introduction

Arterial stiffness is a biomarker for the assessment of cardiovascular disease, and increases along with increasing ages both in healthy young adult and hypertensive patients (Laurent et al., 2012). Generally, thickening, deformation or hardening of the arterial wall will lead to an increase in arterial stiffness, systolic blood pressure and impaired arterial buffering function. Therefore, arterial health indicators, such as systemic arterial stiffness, have emerged as being cardiovascular disease events and all-cause mortality (Laurent et al., 2001; The Reference Values for Arterial Stiffness’ Collaboration, 2010; Karras et al., 2012)–(Laurent et al., 2001; The Reference Values for Arterial Stiffness’ Collaboration, 2010; Karras et al., 2012).

Studies by authoritative institutions have proved that aerobic exercise training is beneficial to reduce arterial stiffness and recommended as the preferred exercise method to prevent and treat cardiovascular disease (American College of Sports Medicine et al., 2018; Williams et al., 2007). While improvements in muscle fitness are known to reduce mortality and blood pressure (Artero et al., 2011; Ruiz et al., 2008). A cross-sectional study reported that young men with strength training showed stiffer arteries than their inactive peers (Miyachi et al., 2003). However, the relationship between resistance training and arterial stiffness progression is still controversial (Li et al., 2015a; Evans et al., 2018). In fact, several studies have shown that arterial stiffness may be increased and decreased as a result of either chronic (Figueroa et al., 2019; Rakobowchuk et al., 2005; Miyachi et al., 2004)–(Miyachi et al., 2004; Rakobowchuk et al., 2005; Figueroa et al., 2019)or acute (Li et al., 2015b; Nitzsche et al., 2016)exposure to traditional resistance training (TRT) for healthy young and middle-aged adults. Besides, a recent meta-analysis (Ceciliato et al., 2020) and study (Werner et al., 2019) suggested that long-term resistance training did not seem to result in any changes in arterial stiffness in young adults. There is strong evidence that TRT is the most effective measure to enhance muscular strength and promote the development of health (Pollock et al., 2000; Suchomel et al., 2018), which is negatively associated with the occurrence of cardiovascular diseases (Artero et al., 2012) and positively with life prospects (García-Hermoso et al., 2018). Thus, although TRT is an effective training method to improve skeletal muscle mass and function and enhance the quality of life (Pollock et al., 2000), more studies are needed to determine its role on arterial stiffness.

The exercise training has been recognized as an effective and non-drug intervention for preventing aging-related cardiovascular events. However, there is few studies assessing the long-term effects of resistance training on cardio-ankle vascular index (CAVI) in untrained young men. Recently, functional resistance training (FRT) has been reported to elicit the health promotion and rehabilitation in patients and elder adults. FRT uses strength exercises generally characterized by synchronized, multiple joint, multiple planes, and unstable movements (e.g., BOSU ball, Swiss ball) that attempt to train muscles, despite with a lower training intensity (La Scala Teixeira et al., 2017), which could achieve the similar effect as to TRT (Vasconcelos et al., 2020; de Resende-Neto et al., 2021). This type of training increased muscle activation when compared to traditional training under stable condition through changes in the surface support. Previous studies well-indicated efficiency of FRT for physical fitness (Feito et al., 2018; Xiao et al., 2021) and capabilities (La Scala Teixeira et al., 2017). Nevertheless, the unknown if the FRT protocols would generate changes in arterial stiffness, and the effect of FRT with unstable condition on muscular fitness has not yet been demonstrated.

In this study, we aimed to investigate the changes in systemic arterial stiffness and muscular fitness components, using criterion-standard techniques, in response to TRT and FRT protocols using high-intensity vs. low-intensity in untrained young men. Secondly, we investigated the association between arterial stiffness and muscular fitness components after intervention. We hypothesize that TRT and FRT would elicit decrease in arterial stiffness, and yield increases in muscular fitness.

## Materials and Methods

### Study Design

In this randomized controlled trial, all participants were randomly assigned into two groups of TRT (*n* = 15) and FRT (*n* = 14) with a simple randomization method. The study protocol was submitted to, and approved by, the Capital University of Physical Education and Sports Ethical Committee and conformed to the Declaration of Helsinki concerning the use of human as study participants. The trial registry number was ChiCTR2100048485 at the website of http://www.chictr.org.cn/. All participants were given verbal and signed consent form prior to the study start.

### Participants

**FIGURE 1:**
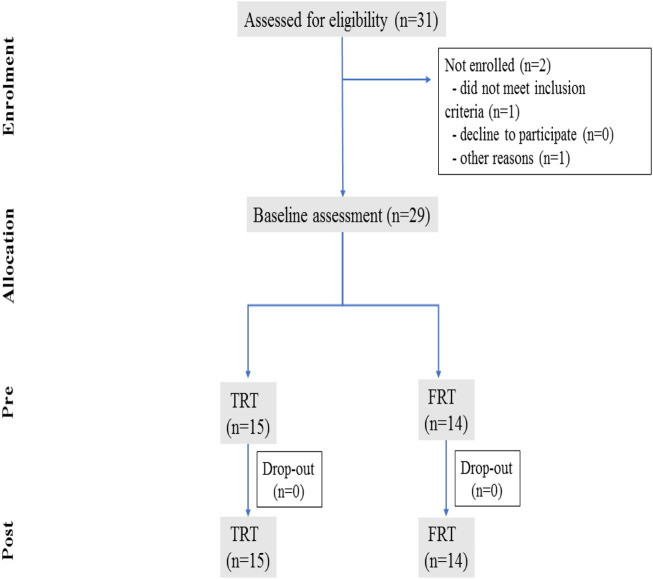
Flow diagram of participants’ training schedule through the study.

All participants were initially screened at the Capital University of Physical Education and Sports in Haidian District, Beijing, China, and were recruited through printed advertisement and by word-of-mouth. Exclusion criteria were current participation in structured regular resistance-type training and aerobic training in last 6 months prior to study initiation, unfavorable cardiovascular disease (e.g., cardiac arrhythmia, peripheral arterial disease), skeletal muscle disease, smokers and alcoholics and use any medication. Thirty-one participants were initially recruited, but two of them were dropped out from the study, as their personal reasons. Consequently, twenty-nine participants (mean age about 22.5 years old) met the recruitment criteria. All participants randomly assigned into two groups of TRT (*n* = 15) and FRT (*n* = 14). The TRT group performed traditional resistance training protocol, while the FRT group participated performed functional resistance training protocol. Finally, twenty-nine untrained young men able to complete all sessions of this study (Figure 1 flow diagram). Table 2 demonstrates the demographic characteristics of two groups.

### Training Protocols

#### Traditional Resistance Training Group and Functional Resistance Training Group Protocols

The participants were trained in six consecutive weeks and three sessions per week. The training protocols consisted of a standard warm-up and cool-down included static and dynamic stretching before and after main training. The whole-body TRT performed five different exercises, including bench press, barbell back squat, deadlift, reverse arm curl and leg flexion under stable surface. The participants external load of training was defined based on their 1-repetition maximum (1RM) tests performed at baseline and middle of the training period. Each session duration was approximately 60 min, exercises were performed in TRT group at 70%1RM, 4–5 sets of 12 repetitions (with 1–2 min rests between each set) to volition fatigue.

The whole-body FRT performed similar training exercises as the TRT group but under unstable surface (using BOSU ball Swish ball, and balance disc). Bench press was performed while lying on the Swiss ball, barbell back squat and dead lift were perform standing on the BOSU ball. Kettlebell swings and Bulgarian split squats were performed on the balance disc. Exercises were performed in FRT group at 40%1RM, 4–5 sets of 20 repetitions, with same rest period between each set. In order to control as many variables as possible, the training volume of two protocols is remained similar. Regarding the repetitions of the FRT group, which was calculated using the following formula: Repetitions = 70% 1RM (kg) ×TRT group repetition/40%1RM (kg). It would be effective to enhance muscle strength in case of new load stimulation. The 1RM tests for all participants were conducted again after 3 weeks of training to make ensure that all participants readjusted training intensities based on their strength gains. To avoid physical activity and diet-induced variability in muscular fitness and arterial stiffness, all participants were asked to maintain normal living habits and avoid attending any extra raining, including aerobic training and any other type of physical activity, and not use any nutritional supplements and drugs during training period.

### Blood Pressure Assessments

Participants were asked to seat quietly for 15 min prior to the formal measurement. Blood pressure (systolic and diastolic pressure) and heart rate were assessed twice in the supine position using the electrocardiogram module of VaSera device. The average of two readings will be represented participants’ final blood pressure for further analysis.

### Arterial Stiffness Assessment

Measurement of cardio-ankle vascular index (CAVI) was conducted according to the newest published principles (Sun, 2013; Shirai et al., 2006; Shirai et al., 2011)–(Sun, 2013; Shirai et al., 2006; Shirai et al., 2011). Based on the beta index, measured using the VaSera device (VS-1500AE/AN, Fukuda Denshi, Tokyo, Japan), which has been recorded at the origin of the aorta to the ankle, and successfully applied in young men in previous study (Li et al., 2015b). All participants were informed to restrain the intake of any supplements (e.g., caffeine and beverage) for at 6 h before testing and 12 h from any type of strenuous exercises. Post-assessments were carried out after 24 h following the completion of the last training session. Prior to participating in this study, participants were properly informed that all measurements were not harmful to their health. Initially, the participants were instructed to seat quietly in a seated position and keep 15 min rest at least in a comfortable room (temperature between 23–25°C), after which four standard pressure cuffs was around the left and right upper arms and ankles in the supine position, and electrocardiogram electrodes linked to the wrist, with a cardiac sonography microphone was fixed on the middle sternum to the left for phonocardiography. As described in the principle, CAVI was calculated automatically by using a vascular screening system (VaSera1500). The main formula used in its system is based on Bramwell Hell equation (Equation 1). CAVI lower than 8 was considered normal, 8–9 was classified as borderline of arterial stiffness, and higher than 9 was consider as suspected arterial stiffness (Thitiwuthikiat et al., 2017). Equation 1 $\mathrm{CAVI}=2\rho ÷\left(\mathrm{Ps}-\mathrm{Pd}\right)\times \ln\left(\mathrm{Ps}÷\mathrm{Pd}\right)\times \mathrm{PW}V^{2}$ Where ρ is represented blood density, *P*s and *P*d are represented systolic blood pressure and diastolic blood pressure, and PWV is pulse wave velocity. The average CAVI was calculated from the both left and right cardio-ankle vascular indices. Then, the average CAVI was obtained and its delta changes (posttest–pretest) in each participant were finally included in the later analysis.

### Muscular Fitness Testing

Muscular fitness components were included 1 repetition maximal strength (bench press, barbell back squat, and dead lift), muscular endurance (70%1RM of bench press repetitions, 70%1RM of leg flexion repetitions), and power (Counter Movement Jump, CMJ). We will demonstrate all testing procedures with more specific details in the following.

### 1 Repetition Maximal Strength Assessment

The 1RM test was assessed consecutively using standard testing procedures before and after 6 weeks training intervention, including bench press, barbell back squat, dead lift and dominant leg flexion. According to the prescription and guidelines of the American College of Sports Medicine (American College of Sports Medicine et al., 2018) suggested that participants gradually increased the load lifted until failure, the highest load completed was recorded as 1RM. Initially, the participant warmed up for 5 min on a paddle ergometer at a perceived exertion level 3 (on the CR 10 Borg scale) followed by two sets of 5–10 repetitions warm-up with 40–60%1RM. On the last set, participants performed 3–5 repetitions at approximately 60–80%1RM. A 1–2 min rest period was allowed between warm-up sets. After the last set, a 3 min rest was taken before the actual 1RM test. Participants attempted to completed through approximately five trials, with the rest period between each trial being approximately 3 min and the highest load achieved in last trial was recorded as the 1RM load. Prior to each strength test, participants were instructed to be familiar with each testing movement pattern. The bench press and barbell back squat, dead lift, and leg flexion exercises were performed on a stable surface. For safety reasons, the 1RM test of the FRT group is also carried out in a stable surface, because the 1RM test in an unstable surface brings a huge risk of injury.

### Muscular Endurance Assessment

Participants were instructed to complete as many as possible for bench press and dominant leg flexion. The same load (70%1RM) was used for pre and post intervention measurements as the previous study suggested (Hackett et al., 2021). According to 1RM test, participants were asked to achieve a full range of motion and proper technique. The repetition cadence was performed in 1 s eccentric and 1 s concentric contraction. The maximal number of repetitions achieved for each exercise was recorded for statistical analysis.

### Power—Counter Movement Jump

The Quattro Jump System (Kistler 9290AD, Switzerland) was used. All participants performed a counter movement jump (CMJ) test without arm swing from the portable force plate. Initially, participants stood straight on the force plate with the hands on the hip as the starting position and, when cued by the instructor, he squatted down rapidly to a 90° knee angle position and jumped straight up as high as possible with the hands on the hip consistently. During the ascending phase the participants left the force plate with the lower limbs fully extended and landed on the force plate with the two feet keeping knees straight to ensure that airtime was measured. As suggested by previous authors (Sattler et al., 2012), the best of three consecutive trials, with appropriate rest between each trial, was used as final test result.

#### Statistical Analysis

The sample size estimation of present study is based on a previous study (Ozaki et al., 2013). Considering the effect size *f* ^2^ = 0.30, the power is 0.80 and the significance level is 0.05 (Cohen, 1992), a minimum number of sample size of 24 (12 in each group) was found to be sufficient using repeated measures within–between interaction (G*Power 3.1; Heinrich Heine, Dusseldorf, Germany). Continuous data were presented as mean ± standard deviation (SD) and were initially confirmed to be normally distributed by the Shapiro-Wilk test. Independent samples *t*-test was used to compare difference between the two groups at baseline and following the intervention. To compare the effectiveness of training protocols, two-way mixed ANOVA was performed. Changes in CAVI and muscular fitness components were analyzed using a time (pretest, posttest) x group (TRT, FRT) within-between subjects ANOVA with repeated measures. A significant time x group interaction was considered to indicated a significant training effect. the independent and paired *t*-test was used to determine simple effects. Furthermore, mean difference in delta value (pretest–posttest) for the two groups are calculated, and the effect sizes were reported as partial eta square converted to Cohens d. A Cohens d from 0–0.2 was considered small, 0.2–0.8 medium, and higher than 0.8 large (Cohen, 1992). Additionally, we used Pearson correlation coefficient for estimating correlations between delta value of arterial stiffness and muscular fitness components (posttest-pretest). All data were analyzed using SPSS 24.0. Value of *p >* 0.05 denotes statistical significance.

## Results

### Participants

Twenty-nine untrained young men (mean age about 22.5 years old) were eligible for this study. Figure 1 shows flow of participants through the study. Two men were excluded for personal reasons. All participants were randomized into the TRT group (*n* = 15) and FRT group (*n* = 14). Baseline participants characteristics are shown in Table 1. There were no differences between the groups on age, height, weight, and body mass index. Additionally, no training-related injuries occurred to any of the participants in two groups, and all participants in both groups successfully completed 18 training sessions during 6 weeks period with no participants dropout.

**TABLE 1:** Group characteristics at baseline as Mean ± SD.

Group	TRT Group	FRT Group	*p* value
Age (years)	22.13 ± 2.94	20.93 ± 2.70	0.261
Height (cm)	176.60 ± 5.45	176.71 ± 5.98	0.958
Weight (kg)	77.95 ± 11.61	73.36 ± 10.20	0.268
Body mass index (kg/m^2^)	24.94 ± 3.12	23.42 ± 2.61	0.168

TRT, traditional resistance training group; FRT, functional resistance training group.

### Arterial Stiffness and Muscular Fitness

The outcomes before and after the resistance training for variables by two groups were shown in Table 2. A mixed ANOVA analysis indicate no significant differences in time x group interaction between CAVI, maximal strength, muscular endurance, and power (*p* > 0.05). However, a significant main time effects were found in these variables (*p* < 0.05), with a small to medium effect size in all of them (Cohens d = −0.20–0.52). Post hoc comparisons for time indicated that a significant decrease in CAVI in FRT group, and a significant increase in maximal strength, muscular endurance, and CMJ were observed in both groups. Additionally, the independent *t*-test exhibited no significant in the reduction of CAVI between the groups.

**TABLE 2:** Comparison of arterial stiffness, blood pressure, heart rate and muscular fitness between groups and effect sizes as Cohen d.

Variables	Group	Pre	Post	MD%	P^G^	ES
Brachial SBP (mmHg)	TRT	126.80 ± 11.93	123.73 ± 11.95	−2.42%	0.577	0.00
	FRT	128.29 ± 7.16	125.29 ± 9.09	−2.34%		
Brachial DBP (mmHg)	TRT	71.93 ± 6.17	73.17 ± 5.33	1.71%	0.832	−0.82
	FRT	74.29 ± 6.04	70.50 ± 6.22	−5.10%		
Heart rate (bmp)	TRT	59.00 ± 9.98	59.07 ± 9.92	0.14%	0.590	−0.15
	FRT	64.21 ± 11.35	61.14 ± 11.15	−4.78%		
CAVI	TRT	6.16 ± 0.61	5.69 ± 0.58	−7.79%	0.053	−0.20
	FRT	5.92 ± 0.53	5.35 ± 0.58*	−9.63%		
Bench press (kg)	TRT	75.00 ± 9.82	89.33 ± 10.92**	19.07%	0.590	0.16
	FRT	71.43 ± 10.27	85.89 ± 10.36**	20.30%		
Barbell back squat (kg)	TRT	116.00 ± 19.93	147.50 ± 15.15**	27.16%	0.959	0.01
	FRT	114.29 ± 16.04	148.93 ± 15.09**	30.27%		
Dead lift (kg)	TRT	118.67 ± 21.34	139.00 ± 16.71**	17.13%	0.913	0.52
	FRT	110.00 ± 25.42	129.82 ± 18.36**	18.00%		
Right−leg flexion (kg)	TRT	43.00 ± 6.49	50.67 ± 7.99**	17.91%	0.126	0.41
	FRT	39.29 ± 6.75	49.64 ± 8.87**	26.47%		
Bench press Rep	TRT	19.53 ± 5.51	29.67 ± 6.28**	51.72%	0.374	0.43
	FRT	17.64 ± 5.32	30.00 ± 7.10**	70.29%		
Right-leg flexion Rep	TRT	21.60 ± 5.18	29.73 ± 8.27**	37.50%	0.907	−0.03
	FRT	23.29 ± 9.21	31.14 ± 7.83**	33.05%		
CMJ (cm)	TRT	59.12 ± 9.14	65.85 ± 5.16^**^	11.33%	0.483	−0.17
	FRT	61.27 ± 10.69	66.32 ± 10.35^**^	8.16%		

SBP, systolic blood pressure; DBP, diastolic blood pressure; CAVI, cardio-ankle vascular index; Rep, repetition; CMJ, counter movement jump; TRT, traditional resistance training group; FRT, functional resistance training group; MD%, mean difference%; Post-Pre, P G value of difference between groups, ES effect sizes as Cohen d, main time effect *p < 0.05, **p < 0.01

**FIGURE 2:**
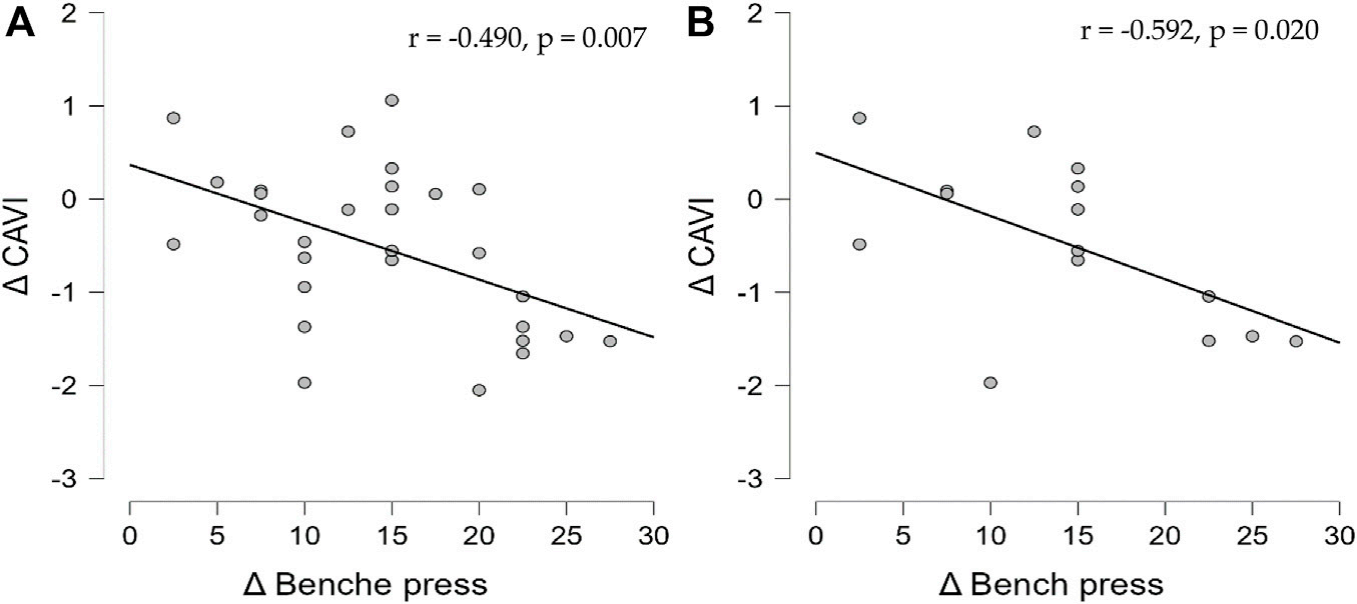
Association between delta value in CAVI and delta value in bench press. **(A)** In all participants, the decrease in CAVI was significant negative correlation with the increase in 1RM bench press. **(B)** In TRT group, the decrease in CAVI was also significantly negative correlated with the increase in 1RM bench press.

The Table 3 show the Pearson correlation coefficients for associations between delta value in CAVI and delta value 1RM bench press. In all participant, there was a significant negative correlation between the decrease in CAVI and the increase in bench press (*r* = −0.490, *p* < 0.01, Figure 2A). In TRT group, the decrease in CAVI was also significantly negative correlated with the increase in 1RM bench press (*r* = −0.592, *p* < 0.05, Figure 2B). However, in FRT group, there was no significant correlation between the delta value in CAVI and the delta value of any muscular fitness variables.

**TABLE 3:** Correlations of delta value in CAVI with muscular fitness components.

Variables	Total		TRT Group		FRT Group	
	*r*	*P*	*r*	*P*	*r*	*P*
Bench press (kg)	−0.490	0.007**	−0.592	0.020*	−0.350	0.220
Barbell back squat (kg)	−0.180	0.350	−0.425	0.114	0.156	0.593
Dead lift (kg)	−0.083	0.668	−0.266	0.337	0.109	0.709
Right-leg flexion (kg)	0.216	0.262	0.360	0.188	0.160	0.584
Bench press Rep	−0.202	0.292	−0.202	0.469	−0.191	0.514
Right-leg flexion Rep	0.026	0.894	−0.051	0.855	−0.536	0.958
CMJ (cm)	−0.149	0.440	−0.032	0.910	−0.563	0.058

Rep, repetition, CMJ, counter movement jump; TRT, traditional resistance training group; FRT, functional resistance training group, *p < 0.05, **p < 0.01.

## Discussions

The novelty of this study was the uses whole-body traditional high-intensity and functional low-intensity resistance training in untrained young men. The functional low-intensity resistance training result in significant decrease in the average value of CAVI, whereas traditional high-intensity did not. Moreover, there was a negative correlation between the delta value in CAVI and delta value in 1RM bench press for all participants and TRT group. It is indicated that the resistance training could induce change in CAVI is accompanied with the change in muscular strength in untrained young men.

Even though both groups performed the resistance training sessions against the different intensity (70% 1RM and 40% 1RM) and the repetitions were always reached muscular failure, it has some positive effects on muscular strength and arterial stiffness. It was worth noting that the average value of CAVI in TRT group did not decrease significantly, while the FRT group induced a significant decrease after training. These results are consistent with those reported in previous randomized controlled trials that chronic resistance training could improve muscle strength and mass (Arazi et al., 2021; Hamid et al., 2022), and produce significant effects on arterial stiffness in young men (Okamoto et al., 2011; Au et al., 2017). Consequently, the present study suggests that, from a health perspective for cardiovascular health, it could be beneficially to reduce the level of systemic arterial stiffness during functional low-intensity resistance training. Moreover, whether it was traditional high-intensity or functional low-intensity training, the repetitions per set to failure could be effective to enhance strength level.

We hypothesized that the functional low-intensity resistance training would result in reduction in arterial stiffness because of the synthesis and release of endothelium-derived relaxing factor—Nitric Oxide (EDRF—NO), which may improve vascular endothelial function and vascular elasticity (Munir et al., 2008), and have been proposed to have anti-atherosclerotic properties (Moncada et al., 1991). On the other hand, Endothelin-1 is a potent vasoconstrictor peptide, which is produced by vascular endothelial cells and has strong proliferative activity on vascular smooth muscle cells. It has previously been shown that the decrease in system arterial stiffness after acute and chronic resistance training can regulate vascular endothelial function by increasing in NO (Maeda et al., 2006; Munir et al., 2008). Thus, altering the concentrations of plasma endothelin-1 and NO may be a potential mechanism to improve arterial stiffness.

However, there is relatively little information about the effect of resistance training on vascular function, including endothelial function and arterial stiffness. Only one study reported the effect of traditional high-intensity resistance training on arterial function that NO increased significantly after training, the arterial stiffness did not change (Maeda et al., 2006), because the selected measurement indicator was cfPWV instead of CAVI, which might explain why most resistance training cannot change or reduce arterial stiffness.

Currently, the effects of functional low-intensity resistance training on vascular function are not clear. We believed that not only the chronic resistance training enhances the muscle strength and the production of more nitric oxide, but also improves the protein composition of vascular wall, and makes the arterial wall more flexibility; however, this issue remains to be further studied. Regardless of the changes to arterial stiffness during the resistance training protocols, improvements in muscular fitness and vascular endothelial function may result in an additional reduction in the risk of cardiovascular disease. Future studies should pay more attention to the effect of functional resistance training on the endothelium-derived relaxing factor and vascular function in young men.

In addition, we observed that there were higher muscular fitness and lower CAVI in both groups after intervention. From the perspective of the current training protocols, we found different findings from other studies that is whole-body traditional high-intensity resistance training-induced change in CAVI is accompanied with the change in 1RM bench press after 6-weeks resistance training protocols in untrained young men, while the performance of the other exercise was not significantly correlated with CAVI. It is widely known that the reduction of arterial stiffness was found after aerobic-based exercise intervention in human being (Ashor et al., 2014; Zhang et al., 2018). However, the influence of strength training or resistance training on arterial stiffness is still controversial. As previous study of strength training intervention found that arterial stiffness did not seem to be affected, but an efficient method to improve muscle function in untrained young men (Werner et al., 2019). Additionally, several months of resistance training “reduces” central arterial compliance (increases central arterial stiffness) in healthy men (Miyachi et al., 2004; Miyachi, 2013). Similarly, baPWV (systemic arterial stiffness) significantly increased after aerobic exercise before high-intensity resistance training (Okamoto et al., 2007). However, in this study, CAVI (systemic arterial stiffness) did not increase and significantly reduced by high-intensity resistance training in young men. From the aforementioned studies, we found that several relevant confounding factors effecting training itself on arterial stiffness, including studies used different training load, samples, and methods to evaluate arterial stiffness. Especially from the measurement indicators, it was not difficult to find that 70–90% of 1RM high-intensity resistance training was performed in these studies. However, authors preferred to use a common indicator named pulse wave velocity (PWV), which has been a widely accepted non-invasive method to the assessment of arterial stiffness, its accuracy is easily affected by changes in blood pressure (Sun, 2013). In recent years, taking the blood pressure into consideration, a novel indicator of CAVI, which is a stiffness and arteriosclerosis indicator of thoracic, independent of arterial blood pressure, was proposed (Shirai et al., 2011). Therefore, it might be feasible to explain the difference results between current and previous studies by using different assessment indicator. In this study, the outcomes confirmed our hypothesis that both two types of resistance training could promote a great improvement on muscular fitness, but only the whole-body functional resistance training could induce a significant reduction in CAVI in untrained young men. Therefore, these findings have important implications to improve arteriosclerosis for the prescription of exercise of young and old adults.

This study has certain limitations that should be considered. Firstly, the improvement of other muscle fitness was also significantly enhanced, but in term of correlation, only bench press was significantly related to arterial stiffness. This might be affected by a small sample size. Hence, the effect of resistance training on arterial stiffness and its correlation with muscular fitness should be further verify in a large sample size in the future. Secondly, daily dietary records should be taken in to account for eliminating difference in calories intake between the two groups which could largely affect the muscular fitness.

## Conclusion

Regarding the findings of this study, using present criterion-standard assessments measurements demonstrates that CAVI was significantly decreased in FRT group after six the weeks resistance training protocol in untrained young men. The reduction in CAVI was negatively correlated with the increasement of 1RM bench press following 6 weeks resistance training intervention. Although the complete mechanism needed to be explored in the future, the current study suggested that cardiovascular health may be regulated by resistance training in untrained young men.

## Data Availability

The raw data supporting the conclusion of this article will be made available by the authors, without undue reservation.
